# A Novel Proximity Biotinylation Assay Based on the Self-Associating Split GFP1–10/11

**DOI:** 10.3390/proteomes8040037

**Published:** 2020-12-02

**Authors:** Aditi S. Kesari, Uma K. Aryal, Douglas J. LaCount

**Affiliations:** 1 Department of Medicinal Chemistry and Molecular Pharmacology and the Purdue Institute of Inflammation, Immunology and Infectious Disease, Purdue University, West Lafayette, IN 47907, USA; aditi.kesari@gmail.com; 2 Bindley Bioscience Center, Department of Comparative Pathobiology and Purdue Proteomics Facility, Purdue University, West Lafayette, IN 47907, USA; uaryal@purdue.edu

**Keywords:** BioID, proximity biotinylation, split-GFP, GFP11, clathrin

## Abstract

Proximity biotinylation was developed to detect physiologically relevant protein–protein interactions in living cells. In this method, the protein of interest is tagged with a promiscuous biotin ligase, such as BioID or BioID2, which produces activated biotin that reacts with nearby proteins; these proteins can subsequently be purified and identified by mass spectrometry. Here we report a novel modification of this technique by combining it with a self-associating split-GFP system in which we exploit the high-affinity interaction between GFP1–10 and GFP11 to recruit BioID2 to the protein of interest. As a test case, we fused GFP11 to clathrin light chain (CLTB) and BioID2 to GFP1–10. Co-expression of GFP11-CLTB and BioID2-GFP1–10 yielded a green fluorescent complex that co-localized with clathrin heavy chain. To facilitate removal of non-specifically biotinylated proteins, we generated an inducible cell line expressing BioID2-GFP1–10. Proximity biotinylation in this cell line with GFP11-CLTB yielded a higher percentage of biologically relevant interactions than direct fusion of BioID2 to CLTB. Thus, this system can be used to monitor expression and localization of BioID bait proteins and to identify protein–protein interactions.

## 1. Introduction

Proximity biotinylation is a promising and increasingly important method to identify protein–protein interactions [1]. In the original version of this approach, the protein of interest was fused to BioID, a mutant biotin ligase from *Escherichia coli*. This mutant enzyme activates biotin, which is then released from the active site rather than being transferred specifically to an acceptor peptide. The resulting cloud of activated biotin quickly reacts with proteins in the vicinity of the protein of interest. The biotinylated proteins are then purified and identified by mass spectrometry. A second-generation version, BioID2, was developed based on a biotin ligase from the thermophilic bacterium *Aquifex aeolicus*, which is smaller, more stable and more effective at identifying protein–protein interactions [2]. 

Proximity biotinylation has several advantages over other approaches for identifying protein–protein interactions. BioID is performed in the cells of interest, which should result in the identification of more physiologically relevant interactions and interactions with proteins that are not properly folded or appropriately modified when expressed in yeast or bacteria. Since interacting proteins are labeled with biotin in intact, living cells, there is less chance for non-specific interactions that may occur after lysis and subsequent mixing of proteins from distinct compartments. Since BioID covalently tags proteins with biotin, weak or transient interactions that would be missed by approaches requiring complexes to be maintained through purification and washing steps can be identified. The covalent linkage also allows harsher wash conditions to be employed, which reduces non-specific binding. 

Although powerful, there are several issues that can present challenges to generating high-quality proximity biotinylation data. The promiscuous nature of the labelling often results in a large number of biotinylated proteins. Rigorous approaches and closely matched negative control proteins are needed to distinguish true interactors from background or non-specifically labeled proteins. In the current version of BioID, it is not possible to monitor expression or localization of the target protein in real time. Finally, the addition of the full-length BioID or BioID2 may not be tolerated by some viruses because large insertions disrupt packaging of the virus genome.

In this study, we sought to address these issues by recruiting BioID to target proteins via a small tag. We considered two small, self-associating peptide tags: GFP11 [3] and HiBiT [4,5]. GFP11 corresponds to the 16 amino acids that comprise the 11th β-sheet of GFP. It spontaneously associates with GFP1–10 (the first ten β strands of super-folder GFP) to generate a green fluorescent complex [3]. This tag has been used to monitor solubility and localization of proteins in living cells [3,6,7]. Similarly, HiBiT is an engineered 13 amino acid peptide that binds to and complements the catalytically inactive LgBiT fragment of nanoluciferase (amino acids 1–156) [4,5]. Both GFP11 and HiBiT bind with similar affinities (~80 pM) to their respective partners [5,8]. Although both systems offer the potential to monitor recruitment of BioID with its target protein via complementation of the self-assembling proteins, we chose to pursue the GFP11 system because it enables easy monitoring of the expression and localization of the protein of interest in the cell population being assayed.

We report here a novel proximity biotinylation system based on the self-assembling split-GFP pair GFP11 and GFP1–10. BioID2 was fused to GFP1–10 and co-expressed with the protein of interest tagged with GFP11. Since localization of clathrin light chain was previously characterized in this split-GFP system and since some of the interacting partners of clathrin light chain are relatively well studied, we tested this system with clathrin light chain tagged with GFP11 [7]. To further improve the removal of non-specifically biotinylated proteins, we established a stable cell line that inducibly expressed BioID2-GFP1–10. We show that complementation of BioID2-GFP1–10 with GFP11 clathrin light chain produced a green fluorescent complex that colocalized with clathrin heavy chain. Proximity biotinylation using this approach yielded a higher quality set of interacting proteins than direct fusion to BioID2.

## 2. Materials and Methods

### 2.1. Plasmids

Plasmids pcDNA3.1-GFP1–10 [7], pEGFP-GFP11-clathrin light chain [7], pMyc-BioID2-MCS [2], and pCAG-FLPo [9] were gifts from Bo Huang (http://n2t.net/addgene:70219; RRID:Addgene_70219; http://n2t.net/addgene:70217; RRID:Addgene_70217), Kyle Roux (Addgene plasmid # 74223; http://n2t.net/addgene:74223; RRID:Addgene_74223), and Philippe Soriano (Addgene plasmid # 13,792; http://n2t.net/addgene:13792; RRID:Addgene_13792), respectively. Plasmid pcDNA3.1 Myc-BioID2-GFP1–10 was constructed by digesting pcDNA3.1-GFP1–10 with *Nhe*I and *EcoR*I and inserting a DNA fragment encoding the Myc tag and BioID2, which was obtained by digesting plasmid Myc-BioID2-MCS. To generate pEGFP-Myc-BioID2-clathrin light chain, Myc-BioID2 was amplified from plasmid Myc-BioID2-MCS using primers (5′-CCAAGCTGGCTAGCCACCATG-3′) and (5′-CGACTGCAGAATTCTCGCTTCTTCTCAGGCTGAACTCG-3′), digested with *Nhe*I and *Eco*RI, and ligated into pEGFP-GFP11-clathrin light chain cut using the same enzymes. To construct pHiBiT-clathrin light chain, oligonucleotides (5′-CTAGCATGGTGAGCGGCTGGCGGCTGTTCAAG AAGATTAGCGGGAGTTCTGGCGGCTCGAGCGGTGGAGCT-3′) and (5′-CCACCGCTCGAGCCG CCAGAACTCCCGCTAATCTTCTTGAACAGCCGCCAGCCGCTCACCATG-3’) were phosphorylated, annealed, and ligated into *Nhe*I- and *Sac*I-digested pEGFP-GFP11-clathrin light chain. pcDNA5/FRT/TO-BioID2-GFP1–10 was constructed by amplifying GFP1–10 from pcDNA3.1-GFP1–10 with primers (5′-CAGCGGCAGTTCTGGCGGTGGATCCATGTCCAAAGGAGAAGAACT GTTTAC-3′) and (5′-CGGGCCCTCTAGACTCGAGCGGCCGCTTATGTTCCTTTTTCATTTGGAT CT-3′), digesting with *Bam*HI and *Not*I, and inserting the purified fragment into plasmid pcDNA5/FRT/TO-BioID2 cut with the same enzymes.

### 2.2. Cell Lines

Human embryonic kidney 293 [HEK293] (ATCC^®^ CRL1573™) cells were purchased from ATCC and maintained in Dulbecco’s modified Eagle’s medium (DMEM) (Gibco™, ThermoFisher Scientific, Waltham, MA, USA) + 10% FBS (R & D Systems, formerly known as Atlanta Biologicals, Minneapolis, MN, USA) at 37 °C in 5% CO_2_. Flp-In™ T-REx™ 293 cells (R78007) were purchased from ThermoFisher Scientific (Waltham, MA, USA) and maintained in DMEM + 10% FBS + 100 μg/mL Zeocin (Gibco™, Waltham, MA, USA). Flp-In™ T-REx™ 293-BioID2-GFP1–10 stable cells were maintained in DMEM + 10% FBS supplemented with 200 μg/mL hygromycin (Gibco™) at 37 °C in 5% CO_2_.

Cells stably expressing BioID2-GFP1–10 were generated as per the manufacturer’s protocol (Invitrogen™, ThermoFisher Scientific, Waltham, MA, USA) with minor changes. Flp-In™ T-REx™ 293 cells were grown in a 6-well plate overnight, then transfected with pcDNA5/FRT/TO-BioID2-GFP1–10 (100 ng) and pFLPo (1 μg); transfection medium was replaced with fresh culture medium after 4 h. On the following day, the cells were transferred into a 10 cm plate and hygromycin B (200 μg/mL) was added 24 h later. The cells were maintained in medium containing hygromycin B for at least two weeks. Medium was changed regularly to remove dead cells. Once colonies were visible, individual colonies were picked, expanded in medium containing hygromycin B and analyzed for protein expression.

### 2.3. Proximity Biotinylation

HEK293 and Flp-In™ T-REx™ 293-BioID2-GFP1–10 stable cells were mock transfected or transfected with plasmids as described in the results section and figure legends using Lipofectamine 2000. For stable GFP1–10 cells, transfection medium was replaced with fresh medium containing 1 μg/mL tetracycline at 3–4 h after transfection. Uninduced, mock-transfected cells were maintained in parallel. At 24 h post-transfection, biotin (50 μM) was added to the culture medium and cells were incubated for 24 h.

### 2.4. Immuno- and Streptavidin Blots

Cells were lysed with 50 mM Tris pH 8, 5 mM EDTA, 150 mM NaCl and 1% Triton X. After collecting cell debris by centrifugation, supernatant was removed and SDS-PAGE sample buffer containing 2% β-mercaptoethanol was added. Electrophoresis was carried out on an SDS-PAGE gel and proteins were transferred to nitrocellulose membranes. BioID2 fusion proteins were detected with chicken anti-BioID2 primary antibody (BioFront Technologies, Tallahassee, FL, USA, product number BID2-CP-100) and IRDye^®^ 800CW donkey anti-chicken secondary antibodies (LI-COR Biosciences, Lincoln, NE, USA, product number 926-32218). Actin was detected with mouse monoclonal anti-actin antibody AC-15 (Sigma-Aldrich, St. Louis, MO, USA, product number A1978) and IRDye^®^ 680RD goat anti-mouse secondary antibodies (LI-COR Biosciences, product number 925-68070). Blots were imaged with an Odyssey^®^ Imaging System (LI-COR Biosciences, Lincoln, NE, USA). Biotinylated proteins were detected with IRDye^®^ 800CW streptavidin (LI-COR Biosciences, product number 925-32230). The original unmodified blots along with the quantitation of the bands are included in Supplementary File 1.

### 2.5. Fluorescent Microscopy

HEK 293 and Flp-In™ T-REx™ 293-BioID2-GFP1–10 stable cells were transfected with GFP expression plasmids as indicated in the text. Flp-In™ T-REx™ 293-BioID2-GFP1–10 stable cells were incubated in the presence of tetracycline to induce BioID2-GFP1–10 expression. Green fluorescence in live cells was imaged using a Zeiss Axio Observer inverted fluorescence microscope equipped with an Axiocam 506 mono camera.

To localize clathrin and BioID2, HEK 293 cells were transfected as described above. On the following day, cells were fixed with 4% paraformaldehyde and permeablilized with 1% Triton X-100. Clathrin was detected by sequentially probing with rabbit anti-clathrin heavy Chain (Abcam, Cambridge, UK, product number ab21679) primary antibodies and Alexa Fluor 594 goat anti-rabbit IgG secondary antibodies (Invitrogen, ThermoFisher Scientific, Waltham, MA, USA, product number A11012). BioID2 was detected with chicken anti-BioID2 primary antibody and Alexa Fluor 488 goat anti-chicken IgG secondary antibodies (Invitrogen, ThermoFisher Scientific, Waltham, MA, USA, product number A11039). Cells imaging was performed using a Nikon A1 Confocal System on Nikon Eclipse Ti Microscope.

### 2.6. Purification of Biotinylated Proteins

HEK 293 cells were plated in T75 flasks and mock transfected or transfected with pcDNA3.1 Myc-BioID2-GFP1–10, pEGFP-Myc-BioID2-clathrin light chain or pEGFP-GFP11-clathrin light chain plus pcDNA3.1 Myc-BioID2-GFP1–10 plasmids in triplicate. At 24 h after the transfection, biotin (50 μM) was added to the cells. After 24 h, cells were washed gently with PBS, flushed from the surface of the flask with fresh PBS, and collected by centrifugation at 500× *g*. Cells pellets were frozen on dry ice and stored at −80 °C.

Triplicate cultures of T-REx™ 293-BioID2-GFP1–10 cells were mock transfected or transfected with pEGFP-GFP11-clathrin light chain or pEGFP-HiBiT-clathrin light chain followed by induction with tetracycline. At 24 h after the transfection, 50 μM biotin was added and cells were processed as described above at 48 h. 

Biotinylated proteins were purified according to the protocol of Hesketh et al. with slight modifications [10]. Cell pellets were weighed, thawed on ice and resuspended at a 1:4 wt:vol ratio in BioID Lysis buffer (50 mM Tris pH7.5, 150 mM NaCl, 0.4% SDS, 1% IGEPAL, 1.5 mM MgCl_2_, 1 mM EGTA) supplemented with 1X Protease Inhibitor mix (Sigma-Aldrich, St. Louis, MO, USA, product number S8830) and 250 U Benzonase (MilliporeSigma, St. Louis, MO, USA, product number 712053) per mL of Lysis Buffer. Cells were frozen on dry ice and immediately thawed at 37 °C. As soon as the cells started thawing, cells were placed on ice and transferred to an end-over-end mixer for 30 min at 4 °C. Cell debris was removed by centrifugation at 21,130× *g* for 20 min. The supernatant was transferred to a fresh tube, to which was added 35 μL of streptavidin beads that were washed three times with BioID Lysis Buffer. The mixture was incubated overnight at 4 °C with rotation using an end-over-end mixer. Beads were collected by centrifugation at 500× *g* for 2 min and washed once with BioID Lysis Buffer, once with BioID Wash Buffer (2% SDS, 50 mM Tris, pH 7.5), twice with BioID Lysis Buffer and three times with 50 mM ammonium bicarbonate solution, pH 8.0. The beads were suspended in ammonium bicarbonate solution and stored at −80 °C.

### 2.7. On Bead Digestion

The bead suspension was thawed on ice and the beads were collected by centrifugation. After removing the supernatant, the beads were resuspended in 10 μL of 8 M urea, 10 mM dithiothreitol (DTT) and incubated for 1 h at 37 °C for reduction. Alkylation was performed by adding 2% (*v*/*v*) of alkylating solution (97.5% acetonitrile, 0.5% triethylphosphine, and 2% iodoethanol) and incubating for 1 h at 37 °C. After drying by vacuum centrifugation, the proteins were digested using 80 μL (0.05 μg/μL) of sequence grade Lys-C/Trypsin (Promega, Madison, WI, USA) in a Barocycler NEP2320 (Pressure Biosciences, Inc., Boston, MA, USA) at 50 °C under 20,000 psi for 1 h. The resulting peptides were cleaned and recovered from the beads using C18 spin columns (Nest Group), dried by vacuum centrifugation, and resuspended in 97% purified water/3% acetonitrile (ACN)/0.1% formic acid (FA).

### 2.8. LC–MS/MS Data Collection and Data Analysis

Mass spectrometry was performed using a Dionex UltiMate 3000 RSLC Nano System coupled to a Q Exactive™ HF Hybrid Quadrupole-Orbitrap Mass Spectrometer (Thermo Scientific, Waltham, MA, USA). Peptides were loaded onto a 300 µm x 5mm C18 PepMap™ 100 trap column and washed for 5 min with 98% purified water/2% ACN/0.1% FA at a flow rate of 5 µl/minute. After washing, the trap column was switched in line with a 75 µm × 50 cm reverse-phase Acclaim™ PepMap™ RSLC C18 analytical column heated to 50 °C. Peptides were separated using a 120 min linear gradient at a flow rate of 0.3 µL/min. Mobile phase A consisted of 0.1% FA in purified water while mobile phase B was 0.1% FA in 80% ACN. The method began at 2% B and reached 10% B in 5 min, 30% B in 80 min, 45% B in 93 min, and 100% B in 93 min. The column was held at 100% B for 5 min before being brought back to 2% B and equilibrated for 20 min. Samples were injected into the QE-HF through the Nanospray Flex™ Ion Source using an emitter tip from New Objective (Littleton, MA, USA). MS data were collected between 400 and 1600 *m/z* using 120,000 resolution at 200 *m/z*, 100 ms maximum injection time, and 15 s dynamic exclusion. The top 20 precursor ions were fragmented by higher energy C-trap dissociation (HCD) at a normalized collision energy of 27%. MS/MS spectra were acquired using the Orbitrap at a resolution of 15,000 at 200 *m/z* and a maximum injection time of 20 ms. 

LC–MS/MS RAW data files were converted into MGF in Mascot Daemon (ver 2.5.1.) using ProteoWizard RAW data import filter. MS/MS spectra were searched against Uniprot human protein database downloaded on 18 November 2018 in Mascot Daemon. To control the FDR (False Discovery Rate), spectra were searched against the corresponding reverse sequence database by selecting a decoy option. The search parameters were as follows: (1) precursor ion (MS1) mass tolerance of 0.05 Da, and product ion (MS/MS) mass tolerance of 0.2 Da, respectively; (2) ethanolyl of cysteine as a fixed modification and oxidation of methionine (M) and acetyl (N-term) as variable modifications, and 1 missed cleavage allowed. Peptide matches were accepted if the significance scores of their match had a *p* value < 0.05. All the matched peptides were filtered to accept peptides with rank 1. The FDR was adjusted to 1% prior to exporting results files. Protein identification generally required a minimum of two peptides and a minimum of one unique peptide if other matched peptides were shared among multiple protein/protein isoforms. Relative protein abundances across samples were determined by spectral counts. The LC-MS/MS RAW data files are available in the MassIVE data repository (massive.ucsd.edu) under ID MSV000086141.

### 2.9. Analysis of BioID Data

Proteins identified by LC–MS/MS analysis were analyzed using the Contaminant Repository for Affinity Purification (CRAPome) at www.crapome.org [11]. Spectral counts from biotinylated proteins identified in clathrin light chain samples were compared to samples from controls cells expressing Myc-BioID2-GFP1–10 using default SAINTexpress parameters (Incorporate Known Data = none, Number of Replicates Per Bait = all) to calculate fold change, SAINT score and Bayesian false discovery rate (BFDR) [12,13]. For purposes of comparison, we report all interactions with a BFDR ≤ 0.02 (high confidence) and BFDR ≤ 0.2 (low confidence). Proteins with BFDR > 0.2 were excluded. 

Human genes annotated with clathrin-related GO terms were downloaded from Amigo (http://amigo.geneontology.org) on 25 August 2020 [14,15]. The set of human proteins that bind to human clathrin light or heavy chain were downloaded from BioGrid version 3.5.188 [16,17]. High and low confidence interactors from clathrin light chain BioID experiments were queried against the GO and clathrin-interacting protein lists.

## 3. Results and Discussion

### 3.1. GFP11-Clathrin Light Chain Recruits Myc-BioID2-GFP1–10 and Colocalizes with Clathrin Heavy Chain

**Figure 1:**
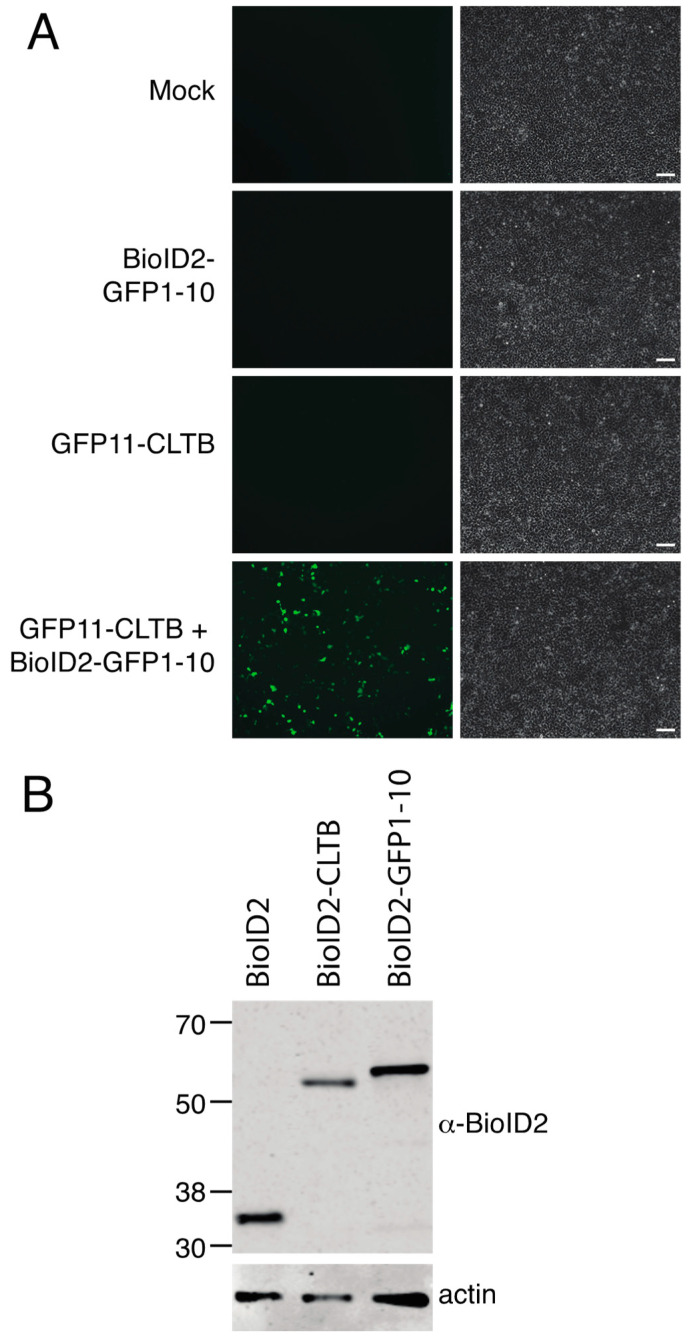
Expression of GFP11-clathrin light chain (CLTB) and BioID2-GFP1–10. (**A**) HEK 293 cells were mock transfected or transfected with plasmids encoding Myc-BioID2-GFP1–10, GFP11-clathrin light chain (CLTB), or Myc-BioID2-GFP1–10 plus GFP11-CLTB. GFP was imaged at 48 h by fluorescence microscopy. Scale bars indicate 100 μM. (**B**) Immunoblot analysis of BioID2 expression. HEK 293 cells were transfected with expression plasmids for Myc-BioID2, Myc-BioID2-GFP1–10, and Myc-BioID2-CLTB. Cell lysates were prepared at 48 h and subjected to SDS-PAGE followed by immunoblotting with anti-BioID2 antibodies. Molecular weight markers are shown to the left of the blot. Predicted sizes are: Myc-BioID2, 31.3 kDa; Myc-BioID2-CLTB, 51.6 kDa; and Myc-BioID2-GFP1–10, 54.3 kDa.

We sought to develop an alternative approach to recruit BioID2 to proteins of interest using a small tag. As a test case, we chose GFP11 as the tag and clathrin light chain as the test protein since GFP11-clathrin light chain was previously reported to complement GFP1–10 and to localize appropriately to clathrin coated pits [7]. We generated an expression construct in which Myc-epitope tagged BioID2 was fused to the amino terminus of GFP1–10. HEK 293 cells co-transfected with GFP11-clathrin light chain and Myc-BioID2-GFP1–10 yielded green fluorescence, whereas cells transfected with each construct individually did not (Figure 1A). Expression of Myc-BioID2-GFP1–10 was confirmed by immunoblotting with anti-BioID2 antibodies (Figure 1B). 

**Figure 2:**
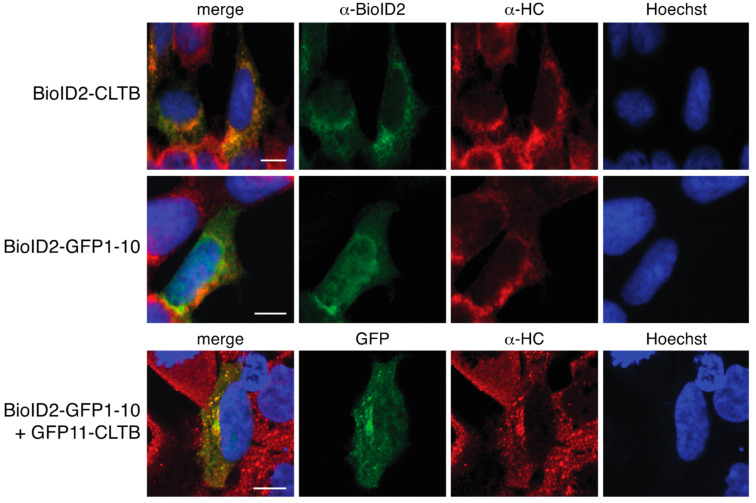
Colocalization of GFP11-CLTB with clathrin heavy chain. HEK 293 cells were mock transfected or transfected with plasmids encoding Myc-BioID2-GFP1–10, Myc-BioID2-CLTB, or Myc-BioID2-GFP1–10 plus GFP11-CLTB. On the following day, cells were fixed, permeabilized and probed with either rabbit anti-clathrin heavy chain and chicken anti-BioID2 primary antibodies (cells expressing Myc-BioID2-GFP1–10 or Myc-BioID2-CLTB) or rabbit anti-clathrin heavy chain primary antibodies only (cells expressing Myc-BioID2-GFP1–10 plus GFP11-CLTB). Primary antibodies were detected with Alexa Fluor 594 anti-rabbit secondary antibodies and Alexa Fluor 488 anti-chicken secondary antibodies. In cells expressing Myc-BioID2-GFP1–10 and GFP11-CLTB, complemented GFP fluorescence was used for localization. Nuclear DNA was labelled with Hoechst 33342 stain. Scale bars indicate 5 μM.

To further evaluate the subcellular localization of GFP11-clathrin light chain, we performed immunofluorescence assays with anti-clathrin heavy chain antibodies. GFP fluorescence overlapped with the signal from clathrin heavy chain, consistent with localization at clathrin-coated pits (Figure 2). In control cells expressing only Myc-BioID2-GFP1–10, the signal was distributed throughout the cell and accumulations suggestive of clathrin coated-pits were not observed (Figure 2). Cells expressing clathrin light chain directly fused to Myc-BioID2 showed a similar overlap with clathrin heavy chain (Figure 2). Thus, GFP11-tagged proteins can recruit BioID2-GFP1–10 to their appropriate subcellular locations.

### 3.2. Identification of Proximity Partners of Clathrin Light Chain in Transiently Transfected Cells

**Figure 3:**
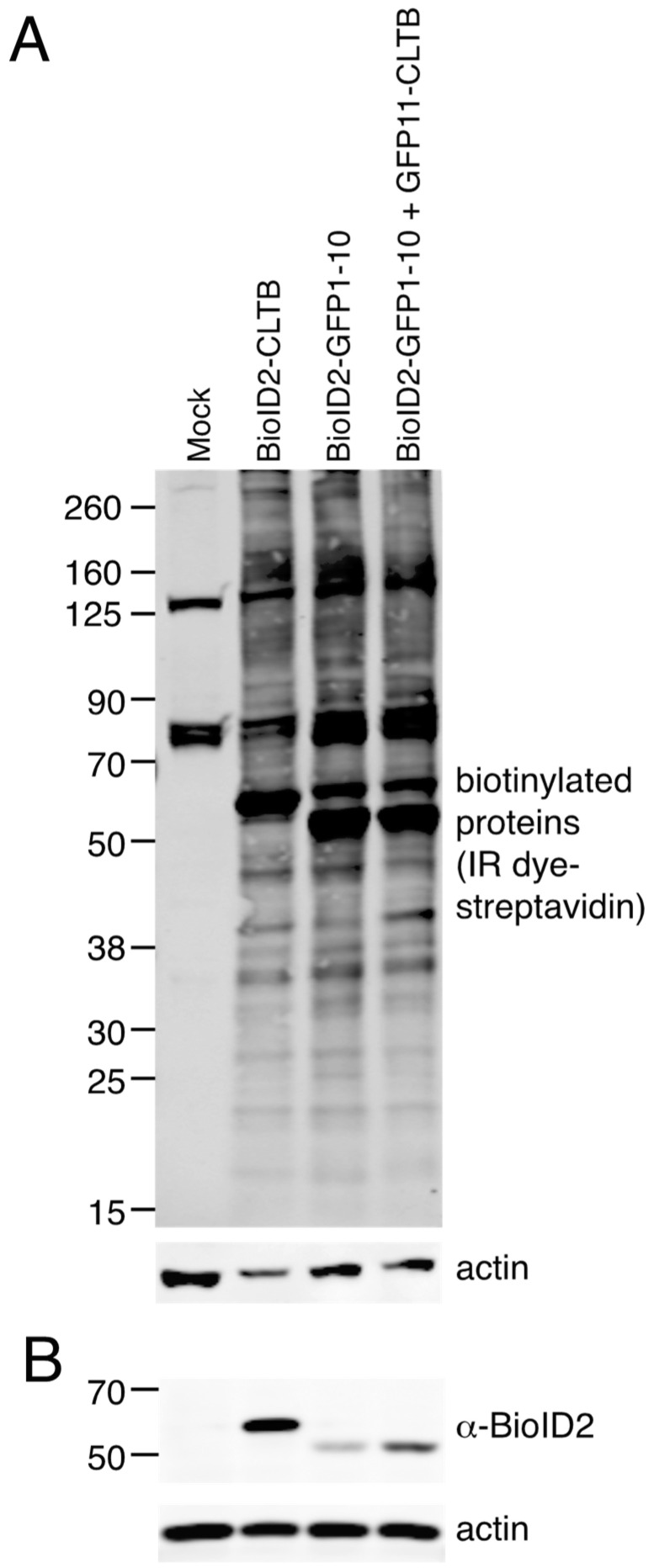
Biotinylation of cellular proteins by Myc-BioID2-GFP1–10 and GFP11-CLTB. (**A**) HEK 293 cells were mock-transfected or transfected with plasmids encoding Myc-BioID2-CLTB, Myc-BioID2-GFP1–10, or Myc-BioID2-GFP1–10 plus GFP11-CLTB, incubated with biotin and lysed. Samples were subjected to SDS-PAGE, transferred to nitrocellulose membrane, and probed with IRDye^®^ 800CW streptavidin. Actin was stained with anti-actin antibodies as a loading control. (**B**) Expression of BioID2 in the samples from panel A was confirmed by probing with anti-BioID2 primary antibodies. Actin was stained with anti-actin antibodies as a loading control.

To identify proteins in proximity to clathrin light chain, HEK293 cells were transiently transfected with plasmids expressing Myc-BioID2-GFP1–10 plus GFP11-clathrin light chain or Myc-BioID2-GFP1–10 alone. For comparison, we transfected cells with a plasmid in which Myc-BioID2 was directly fused to clathrin light chain. Blotting with IRDye-labelled streptavidin revealed extensive biotinylation in samples expressing Myc-BioID2-GFP1–10, with few if any obvious differences in the presence or absence of GFP11-clathrin light chain (Figure 3). Many fewer biotinylated proteins were detected in mock-transfected cells, which represents endogenously biotinylated proteins.

Biotinylated proteins were purified using streptavidin beads and identified by mass spectrometry. Specifically labelled proteins were determined by comparing the spectral counts of biotinylated proteins identified in cells expressing Myc-BioID2-clathrin light chain or co-expressing GFP11-clathrin light chain and Myc-BioID2-GFP1–10 to control cells expressing only Myc-BioID2-GFP1–10 using the CRAPome database [11]. High- (Bayesian false discovery rate (BFDR) ≤ 0.02) and low- (BFDR 0.2–0.02) confidence interactions from GFP11-clathrin light chain and Myc-BioID2-clathrin light chain are listed in Supplementary Tables S1 and S2.

As expected, clathrin light chain protein had the highest spectral counts in samples from cells expressing Myc-BioID2-clathrin light chain or co-expressing GFP11-clathrin light chain plus Myc-BioID2-GFP1–10. Known interacting partners of clathrin light chain, such as clathrin interactor 1 (CLNT1), also were detected. However, only five proteins scored as high-confidence interactors (BFDR ≤ 0.02) in GFP11-clathrin light chain plus Myc-BioID2-GFP1–10 samples, whereas 98 proteins BioID2-clathrin light chain-expressing cells scored as high confidence. Similarly, the total number of biotinylated proteins detected by mass spectrometry was much higher in cells transfected with the BioID2-clathrin light chain construct compared to either GFP11-clathrin light chain plus Myc-BioID2-GFP1–10 or Myc-BioID2-GFP1–10. This mismatch made removing non-specifically biotinylated proteins challenging and prompted us to pursue alternative approaches.

### 3.3. Establishment of a Tetracycline-Inducible, Stable Cell Line Expressing BioID2-GFP 1–10

**Figure 4:**
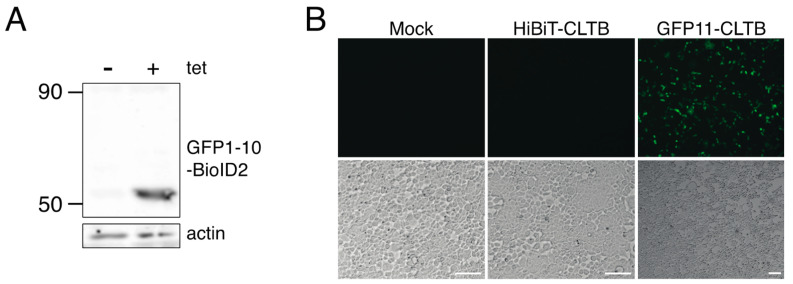
GFP fluorescence complementation in a stable BioID2-GFP 1–10-inducible cell line. (**A**) Flp-In™ T-REx™ 293-BioID2-GFP1–10 stable cells were mock-treated or induced with tetracycline for 24 h. Cells were lysed and immunoblot analysis was performed using with chicken anti-BioID2 primary antibodies. (**B**) Flp-In™ T-REx™ 293-BioID2-GFP1–10 stable cells were either mock transfected or transfected with pEGFP-HiBiT-CLTB or pEGFP-GFP11-CLTB followed by induction with tetracycline. At 24 h after induction, GFP was imaged by fluorescence microscopy. Scale bars indicate 100 μM.

To improve the removal of background biotinylated proteins, we established a stable, inducible cell line that expressed BioID2-GFP1–10 upon addition of tetracycline to the culture medium. The goal of this cell line was to reduce the variation in expression due to co-transfection of multiple plasmids and to ensure that BioID2 was expressed at equal levels in test and control samples. Inducible expression of BioID2-GFP1–10 was confirmed by immunoblotting (Figure 4A). Upon transfection with the GFP11-clathrin light chain expression plasmid and induction with tetracycline, green fluorescent cells were observed (Figure 4B). Green fluorescent cells were not detected in mock-transfected cells or cells transfected with a HiBiT-clathrin light chain expression plasmid following induction with tetracycline. 

### 3.4. Identification of Clathrin Light Chain Proximity Partners in Stable BioID2-GFP 1–10 Cells

**Figure 5:**
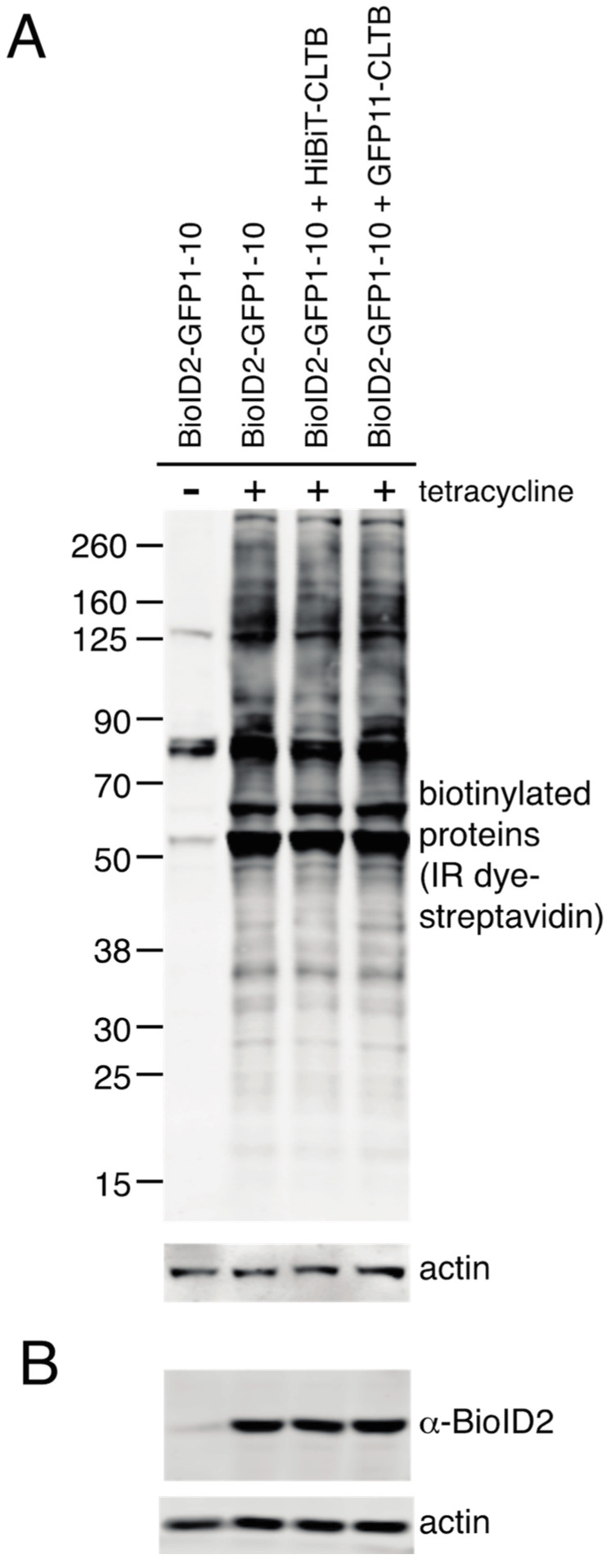
Biotinylation of cellular proteins in stable Myc-BioID2-GFP1–10 inducible cells. (**A**) Flp-In™ T-REx™ 293-BioID2-GFP1–10 stable cells were mock transfected or transfected with plasmids encoding HiBiT-CLTB or GFP11-CLTB and induced with tetracycline. Biotin was added to the culture medium 24 h after transfection and cell lysates were prepared at 48 h. Uninduced, mock-transfected cells were maintained in parallel. Samples were processed as described in the legend to Figure 3. (**B**) Expression of BioID2-GFP1–10 in the samples from panel A was confirmed by probing with anti-BioID2 primary antibodies. Actin was stained with anti-actin antibodies as a loading control.

To identify proximal proteins of clathrin light chain, BioID2-GFP1–10 stable cells were transfected with plasmids expressing GFP11- or HiBiT-tagged clathrin light chain; the HiBiT tag is similar in size to GFP11 but does not bind to GFP1–10. Tetracycline was added to the cells following transfection and biotin was added 24 h later. At 48 h post-transfection, cells were lysed and biotinylated proteins were purified using streptavidin beads. Blotting with IR-Dye streptavidin revealed similar patterns of biotinylation in mock-, GFP11-clathrin light chain, and HiBiT-clathrin light chain transfected cells (Figure 5). In the absence of tetracycline, only a few endogenously biotinylated proteins were observed.

**Figure 6:**
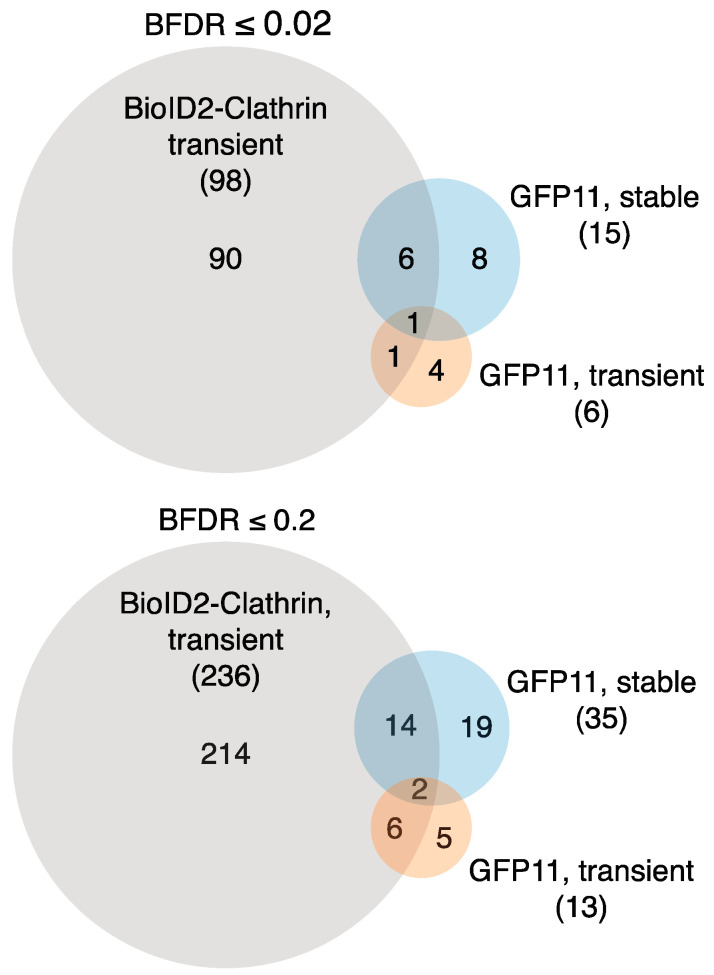
Comparison of BioID datasets. Venn diagrams show the overlap between proteins identified BioID2-CLTB, transient expression of GFP11-CLTB plus BioID2-GFP1–10, and expression of GFP11-CLTB in stable BioID2-GFP1–10 cells. Upper panel shows high-confidence interactions (BFDR ≤ 0.02). Lower panel shows high- and low-confidence interactions (BFDR ≤ 0.2).

Biotinylated proteins were identified by mass spectrometry. To distinguish proteins specifically biotinylated in the presence of GFP11-clathrin light chain from those non-specifically biotinylated by BioID2-GFP1–10, SAINT and BFDR scores were calculated using the CRAPome database with HiBiT-clathrin light chain serving as the negative control (Supplementary Table S3). In comparison to transiently expressed BioID2-clathrin light chain, fewer proteins overall were identified in BioID2-GFP1–10 stable cells expressing GFP11-clathrin light chain and fewer interactions were classified as high or low confidence after SAINT analysis (high confidence, 98 vs. 15 proteins for BioID2-clathrin light chain and GFP11-clathrin light chain plus BioID2-GFP1–10, respectively; low confidence, 236 vs. 35, respectively). About half of the proteins identified in the GFP11 system were also identified in cells expressing clathrin light chain directly linked to BioID2 (Figure 6).

**Figure 7:**
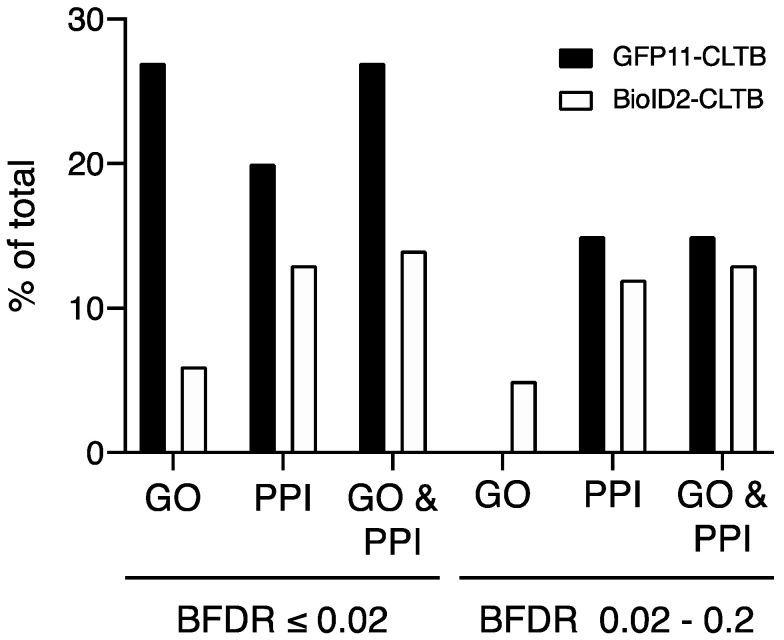
Clathrin-related GO terms and clathrin interacting proteins in the CLTB BioID datasets. The percentage of proteins annotated with “clathrin” Gene Ontology (GO) terms, reported to interact with either clathrin light chain or clathrin heavy chain (PPI) or both is graphed. High- (BFDR ≤ 0.02) and low- (BFDR between 0.02 and 0.2) confidence interactions are shown.

To assess the biological relevance of the proteins identified, we compared the proteins identified by BioID to the list of human proteins annotated with clathrin-related GO terms and to the set of human proteins in the BioGrid database that interacted with either clathrin heavy or light chain. Although transiently expressed BioID2-clathrin light chain identified a larger number of proteins from either group, a higher percentage of proteins identified by GFP11-clathrin light chain were annotated with clathrin GO terms or interacted with clathrin compared to BioID2-clathrin light chain (27 and 20% vs. 6 and 13%, respectively; Figure 7 and Supplementary Table S4). In addition, a larger percentage of proteins with clathrin GO terms or that interacted with clathrin was present in the high confidence set of interactions from GFP11-clathrin light chain than among low-confidence interactions (27% vs. 15%, respectively). In contrast, the same percentage was observed in the high and low confidence sets from BioID2-clathrin light chain (14%). This suggests that non-specific interactions were more effectively filtered out of the highest scoring interactions using the GFP11 fusion approach.

Similar conclusions were reached by Chojnowski et al., who developed an alternative approach, 2C-BioID, to bring BioID to a target protein [18]. In 2C-BioID, BioID2 was fused to FKBP and the target protein was fused to FRB. BioID2 was then recruited to the protein of interest by adding the rapamycin analog AP21967 to induce dimerization of FKBP and FRB. In an analysis of the interactomes of lamin A, lamin C and lamina-associated polypeptide 2b (LAP2b), fusion to FRB enabled proper localization of the target proteins and the identification of a more biologically relevant set of interactions than direct fusion to BioID2. Thus, both GFP1–10/11 and 2C-BioID improve the removal of non-specifically biotinylated proteins, which is the main challenge of BioID. 2C-BioID has the advantage of inducible recruitment of BioID, whereas the GFP11/GFP1–10 system enables the visualization of the target protein in live cells.

This study identified novel clathrin light chain interactions and provided additional support for previously reported interactions. Among the latter category was EDC4, which was first identified as a binding partner of clathrin light chain in a large-scale complex purification plus mass spectrometry study but which has not been characterized in depth [19]. In the present study, EDC4 was identified as a high-confidence interaction with transiently expressed GFP11-clathrin light chain plus GFP1–10-BioID2 and with BioID2-clathrin light chain; it was just above the high confidence cutoff with GFP11-clathrin light chain expressed in stable GFP1–10-BioID2 cells. EDC4 is a component of the mRNA decapping complex and is required for P-body formation [20,21,22]. However, EDC4 also regulates DNA repair through its interaction with BRCA1 and regulates mRNA stability independently of mRNA decapping via its interaction with MARF1 [23,24]. Proximity biotinylation of MARF1 identified the mRNA decapping complex and clathrin, providing independent support for an association of clathrin and EDC4 [25]. In addition to EDC4, several other RNA processing factors were identified, including SF3A1, SF3B1, and PRPF40A, which may suggest unexpected links between clathrin and mRNA processing, or conversely novel roles for RNA binding proteins.

The expression level of the target protein is correlated to the number of biotinylated proteins identified. In the system reported here, expression of BioID2-GFP1–10 cells can be optimized in BioID2-GFP1–10 stable by using different concentrations of tetracycline or by inducing expression for different lengths of time prior to adding biotin. Although it may also be possible to increase the number of protein identified by boosting the level of expression of GFP11-tagged protein, this may lead to improper localization and an increase in non-specific labelling. An alternative is to increase the number of GFP11 tags in order to recruit more BioID to the target protein [7].

## 4. Conclusions

We developed a novel modification of the proximity biotinylation assay by combining BioID2 with a bipartite, self-associating split-GFP system. In doing so, we generated a single approach that can be used to image target protein expression and localization as well as identification of protein–protein interactions. By establishing a stable cell line expressing BioID2-GFP1–10, we created a system for more effective removal of non-specifically biotinylated proteins. Parallel cultures expressing the protein of interest tagged with either GFP11, which binds to GFP1–10 and complements GFP fluorescence, or HiBiT, which is of similar size to GFP11 but does not interact with GFP1–10, enable efficient removal of background biotinylated proteins under nearly identical conditions. The incorporation of the small GFP11 tag is expected to be useful in situations where fusions to the larger BioID protein are not tolerated.

## Supplementary Materials

The following are available online at https://www.mdpi.com/2227-7382/8/4/37/s1, Supplementary File 1: Original western and streptavidin blots and quantitation of bands; Supplementary Table S1: Proteins identified by proximity biotinylation with transiently co-expressed GFP11-CLTB and BioID2-GFP1–10 in HEK 293 cells; Supplementary Table S2: Proteins identified by proximity biotinylation with transiently expressed BioID2-CLTB in HEK 293 cells; Supplementary Table S3: Proteins identified by proximity biotinylation with GFP11-CLTB in stable BioID2-GFP1–10 cells; Supplementary Table S4: Proteins with “clathrin” GO terms or that interact with clathrin heavy or light chain.
