# Supplementary material for: A Novel Proximity Biotinylation Assay Based on the Self-Associating Split GFP1–10/11
Source: Proteomes. 2020 Dec 2;8(4):37. doi: 10.3390/proteomes8040037 (PMC7709110; doi:10.3390/proteomes8040037)

Quantitation of bands on western and IRDye® 800CW streptavidin blots

Two-color image files obtained from a Licor Odyssey CLx Infrared Imaging System were opened in ImageJ and split into red and green channels. Bands on western blots were outlined with a rectangle that encompassed the largest band on the blot. The density of each band was measured using the “Measure” function in ImageJ. To calculate the background signal, the density of an area above or below each band in which no obvious bands were present was measured. Corrected density was calculated by multiplying the mean density of each band by the area, subtracting the average of the background sample, and multiplying by 1000. Relative density was determined by dividing the corrected densities by the corrected density of the most intense band.

The densities of individual bands on IRDye® 800CW streptavidin blots were not calculated due to the sheer number of bands present. Rather, plot profiles of each lane were generated using the “Plot profile” tool in ImageJ. The plot profiles show the intensity of each point along the length of the lane (shown in blue in plot profile graphs). Baseline intensity (shown in black) was measured in the same manner using a line between lanes 1 and 2 on the blots in figures 3A and 5A.

Figure 1 band quantitation

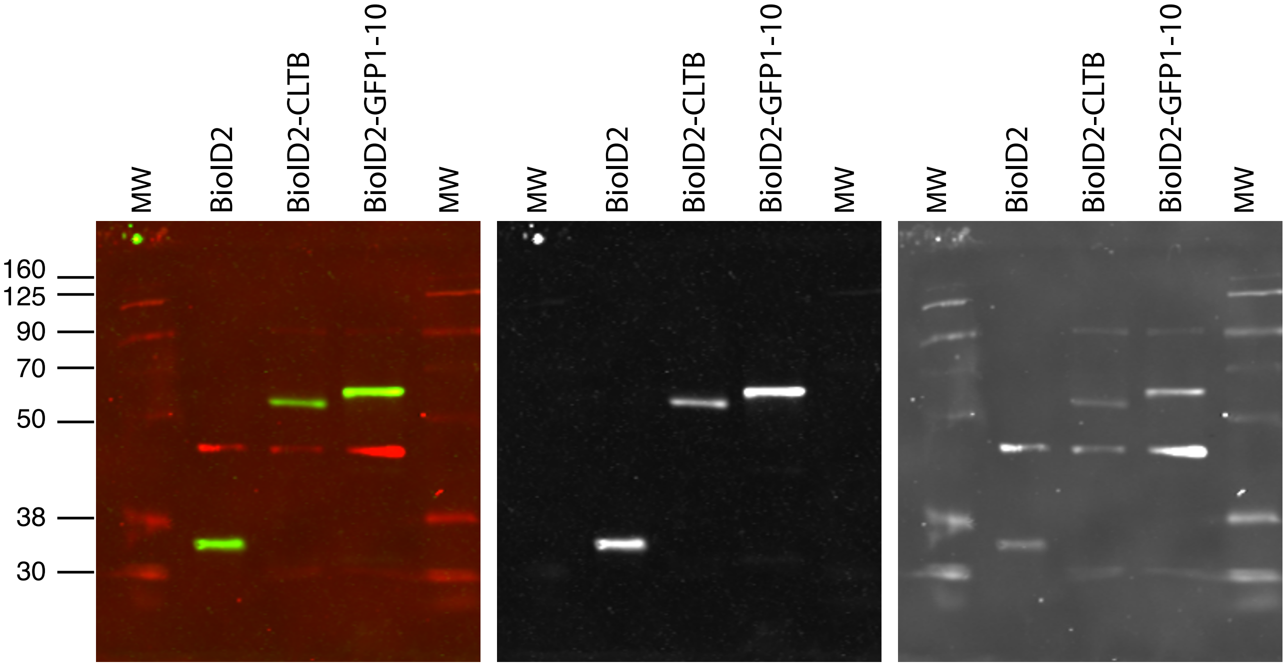

Original two-color image from Licor scanner:
Green = anti-BioID2
Red = anti-actin

Original two color image from Licor scanner
Green layer = anti-BioID2

Original two color image from Licor scanner
Red layer = anti-actin

BioID2 densitometry

Lane	band	Density	Relative density *
1	BioID2	8505	0.78
2	BioID2-CLTB	5157	0.47
3	BioID2-GFP1-10	10,940	1.00

* relative to Lane 3 BioID2-GFP1-10 band

Actin densitometry

Lane	band	Density	Relative density *
1	actin	2630	0.38
2	actin	1173	0.17
3	actin	6846	1.00
1	BioID2 bleed through	1544	0.23
2	BioID2 bleed through	925	0.14
3	BioID2 bleed through	3177	0.46
2	upper band ~90 kDa	296	0.04
3	upper band ~90 kDa	169	0.02

* relative to Lane 3 actin band

Figure 3A band quantitation

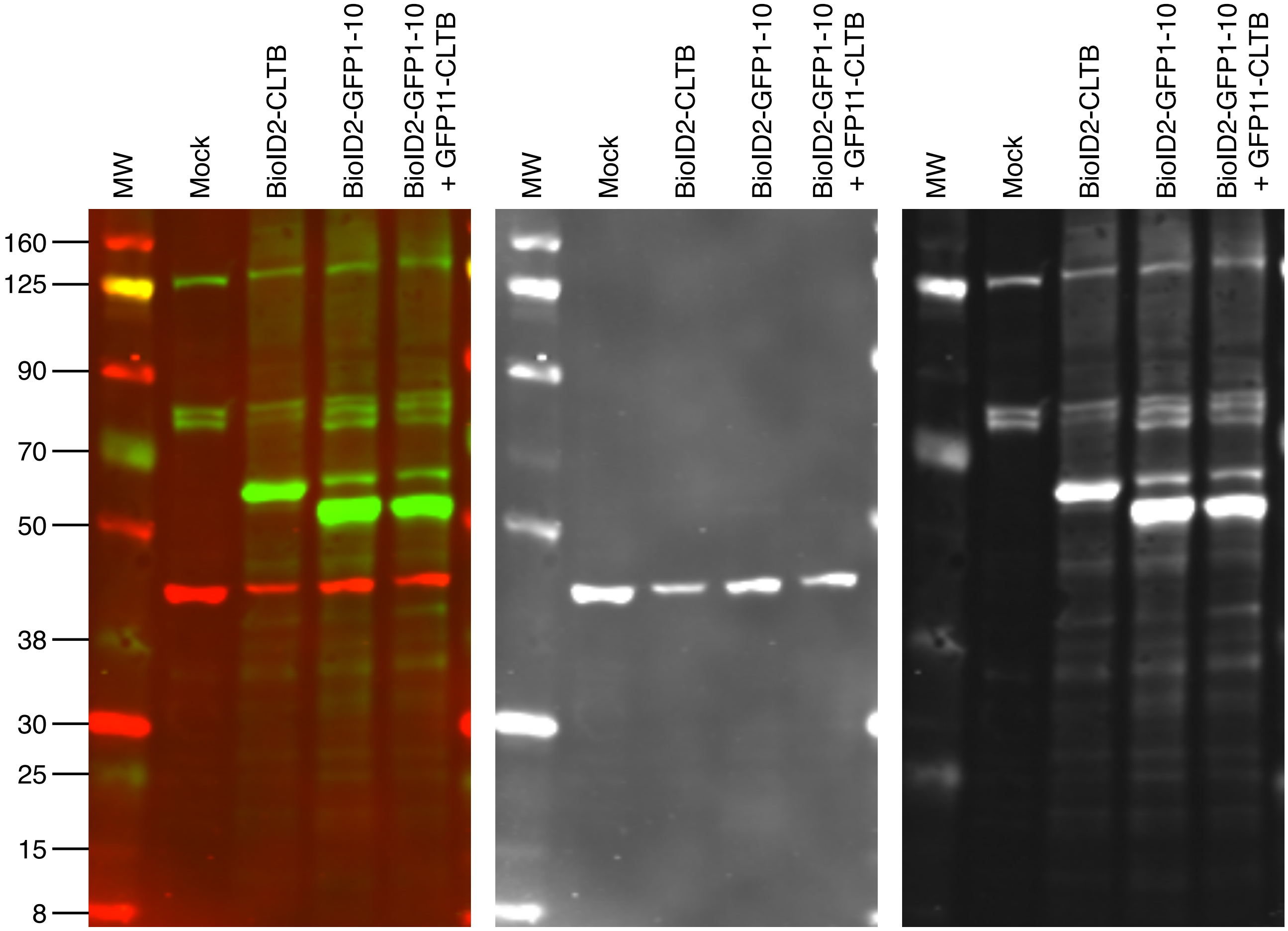

Original two-color image from Licor scanner
Green = anti-BioID2
Red = anti-actin

Original two color image from Licor scanner
Red layer = anti-actin

Original two color image from Licor scanner
Green layer = IRDye® 800CW streptavidin

Actin densitometry

Lane	band	Density	Relative density *
1	actin	12,756	1.00
2	actin	5050	0.40
3	actin	8602	0.67
4	actin	6847	0.54

* relative to Lane 1 actin band

Figure 3A Lane profiles

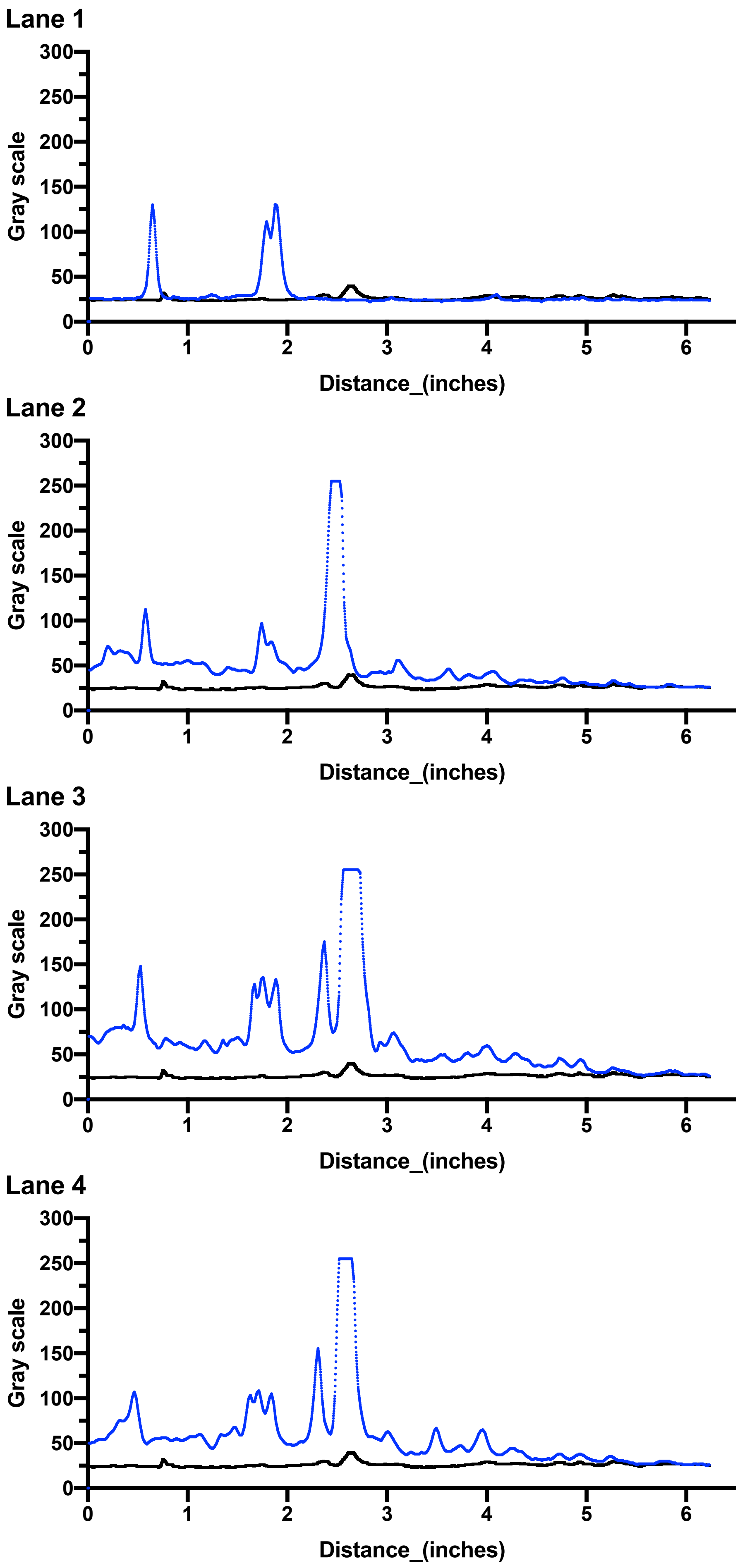

Figure 3B and 5B band quantitation

Original two-color image from Licor scanner: Green = anti-BioID2, Red = anti-actin

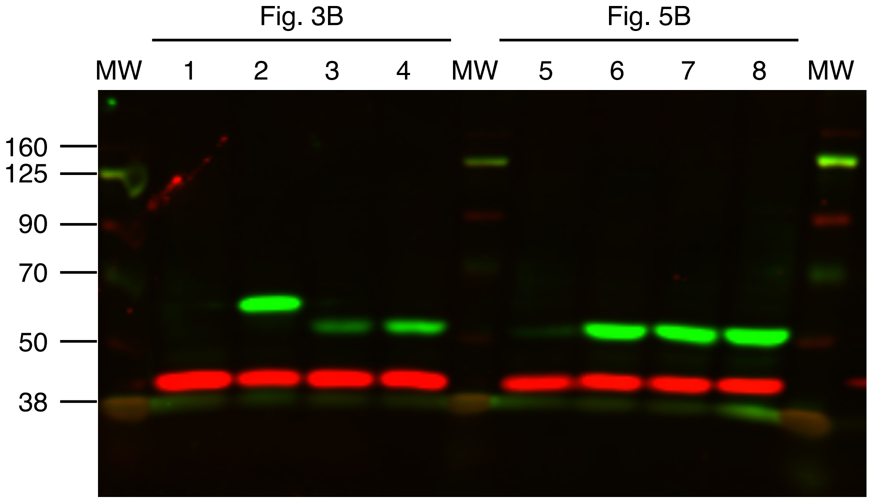

Lane number, sample
1, Mock transfected
2, BioID2-CLTB
3, BioID2-GFP1-10
4, BioID2-GFP1-10 + GFP11-CLTB
5, BioID2-GFP1-10, no tetracycline
6, BioID2-GFP1-10, + tetracycline
7, BioID2-GFP1-10 + HiBiT-CLTB, + tetracycline
8, BioID2-GFP1-10 + GFP11-CLTB, + tetracycline

Original two-color image from Licor scanner with Licor Chameleon duo molecular weight markers overexposed

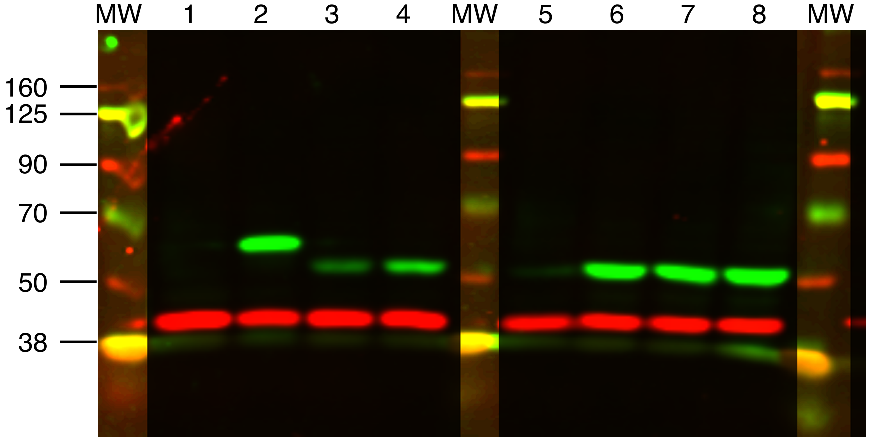

Original two color image from Licor scanner Green layer = anti-BioID2

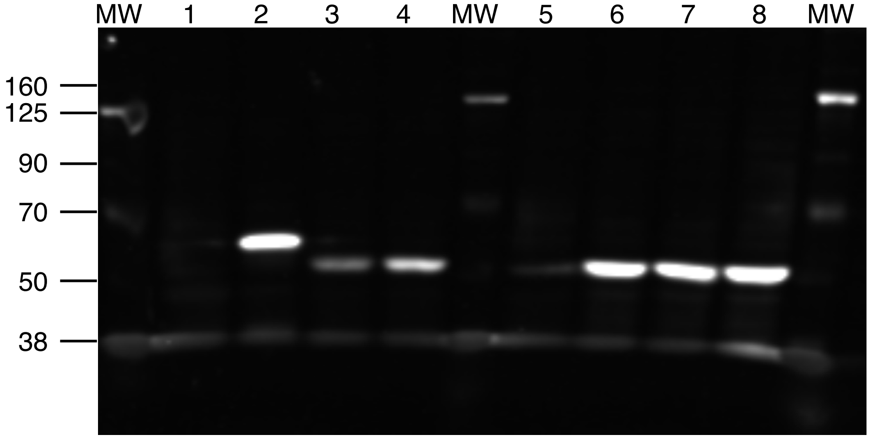

BioID2 densitometry

Lane	band	Density	Relative density *
2	BioID2-CLTB	4214	0.93
3	BioID2-GFP1-10	1196	0.27
4	BioID2-GFP1-10	2511	0.56
5	BioID2-GFP1-10	295	0.07
6	BioID2-GFP1-10	4132	0.92
7	BioID2-GFP1-10	4131	0.92
8	BioID2-GFP1-10	4510	1.00
1	faint band at ~38 kDa	791	0.18
2	faint band at ~38 kDa	302	0.07
3	faint band at ~38 kDa	405	0.09
4	faint band at ~38 kDa	366	0.08
5	faint band at ~38 kDa	401	0.09
6	faint band at ~38 kDa	330	0.07
7	faint band at ~38 kDa	295	0.07
8	faint band at ~38 kDa	1150	0.26

* relative to Lane 8 BioID2-GFP1-10 band

Original two color image from Licor scanner Red layer = anti-actin

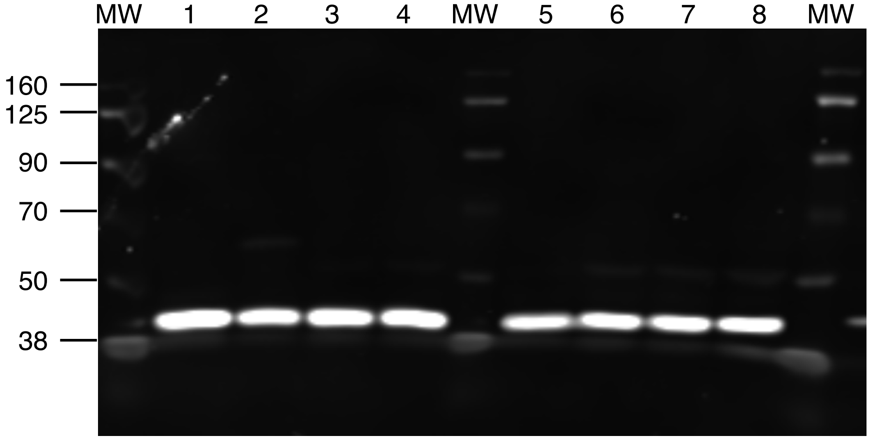

Actin densitometry

Lane	band	Density	Relative density *
1	actin	6499	1.00
2	actin	4420	0.68
3	actin	4928	0.76
4	actin	4922	0.76
5	actin	4295	0.67
6	actin	4357	0.66
7	actin	4148	0.64
8	actin	4239	0.65

* relative to Lane 1 actin band

Figure 4A band quantitation

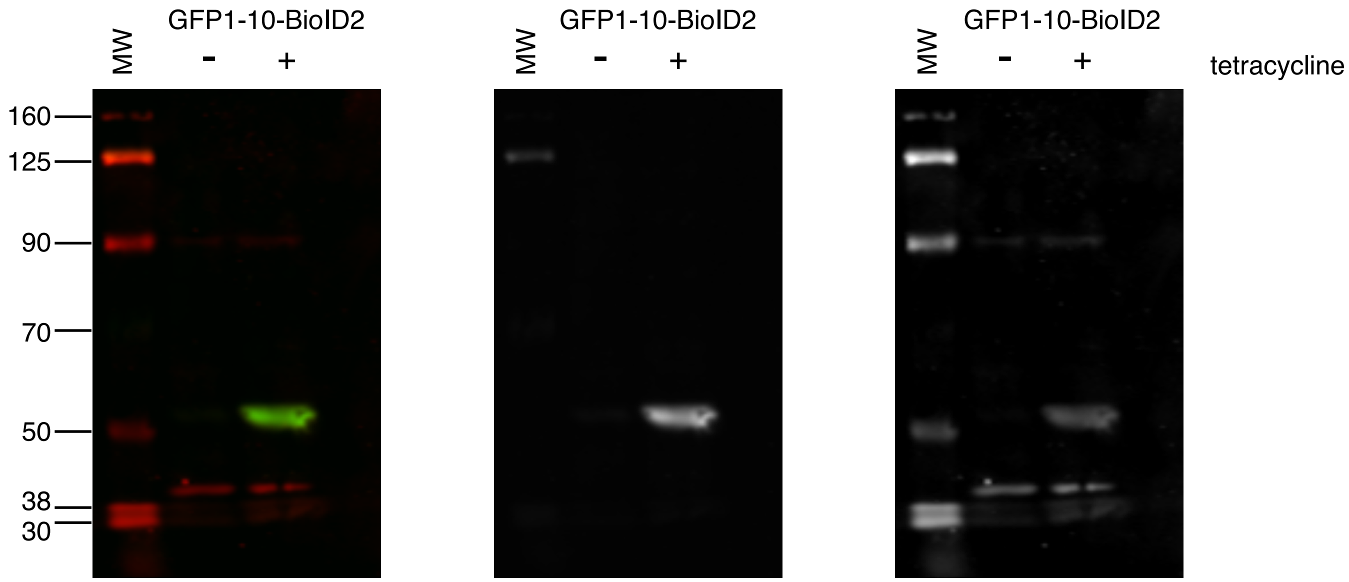

Original two-color image from Licor scanner:
Green = anti-BioID2
Red = anti-actin

Original two color image from Licor scanner
Green layer = anti-BioID2

Original two color image from Licor scanner
Red layer = anti-actin

BioID2 densitometry

Lane	band	Density	Relative density *
]2	BioID2-GFP1-10	4579	1.00

* relative to Lane 3 BioID2-GFP1-10 band

Actin densitometry

Lane	band	Density	Relative density *
1	actin	1164	0.25
2	actin	1020	0.22
2	BioID2 bleed through	2389	1.00
1	upper band ~90 kDa	200	0.04
2	upper band ~90 kDa	402	0.09

* relative to Lane 3 actin band

Figure 5A band quantitation

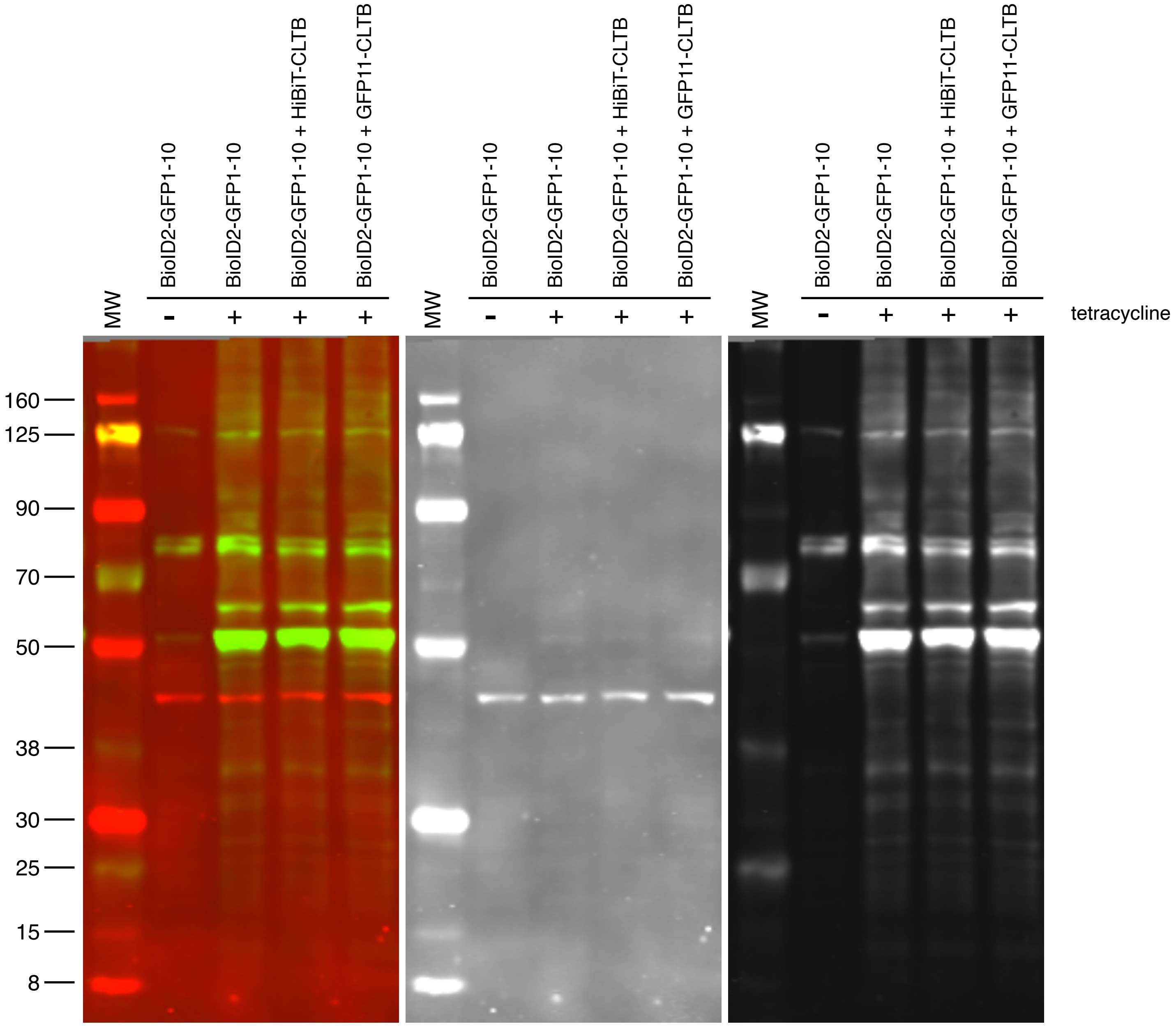

Original two-color image from Licor scanner
Green = anti-BioID2
Red = anti-actin

Original two color image from Licor scanner
Red layer = anti-actin

Original two color image from Licor scanner
Green layer = IRDye® 800CW streptavidin

Actin densitometry

Lane	band	Density	Relative density *
1	actin	3762	0.82
2	actin	3457	0.76
3	actin	3979	0.87
4	actin	4562	1.00

* relative to Lane 4 actin band

Figure 5A Lane profiles

Lane 1

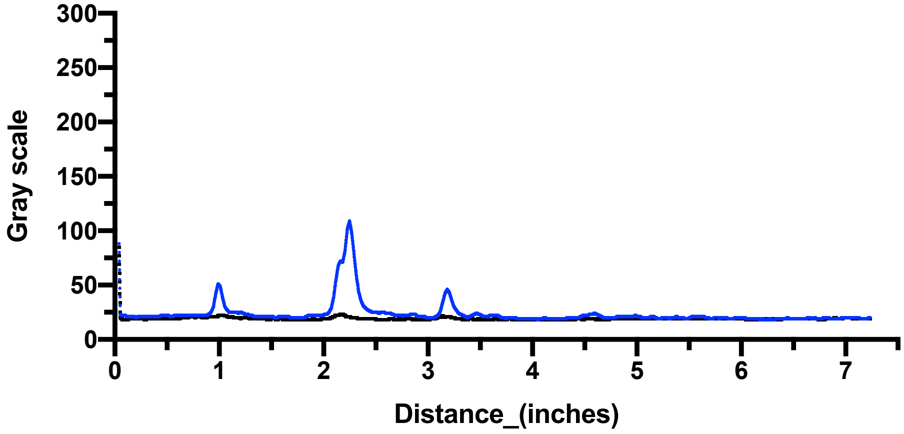

Lane 2

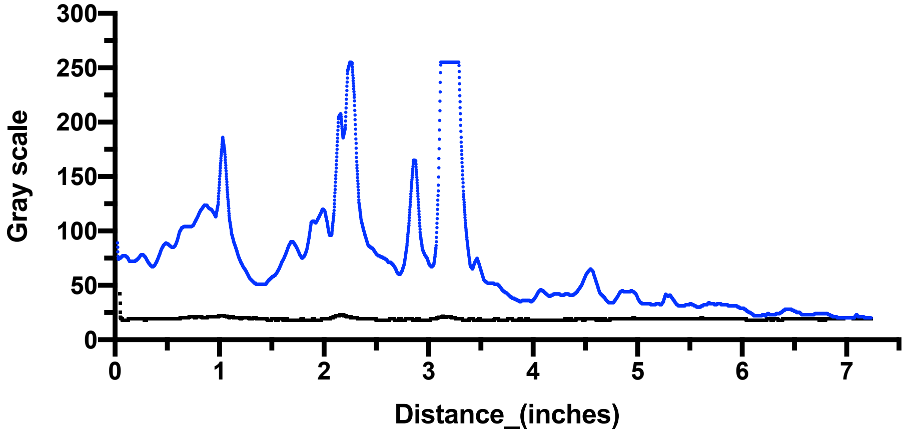

Lane 3

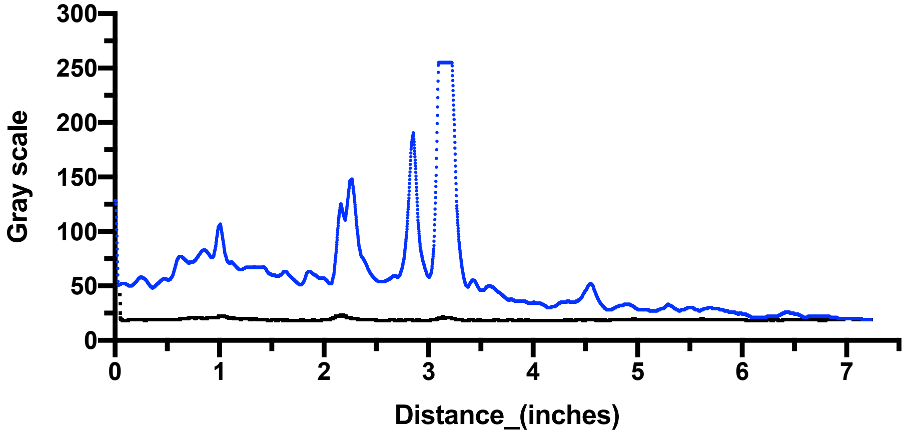

Lane 4

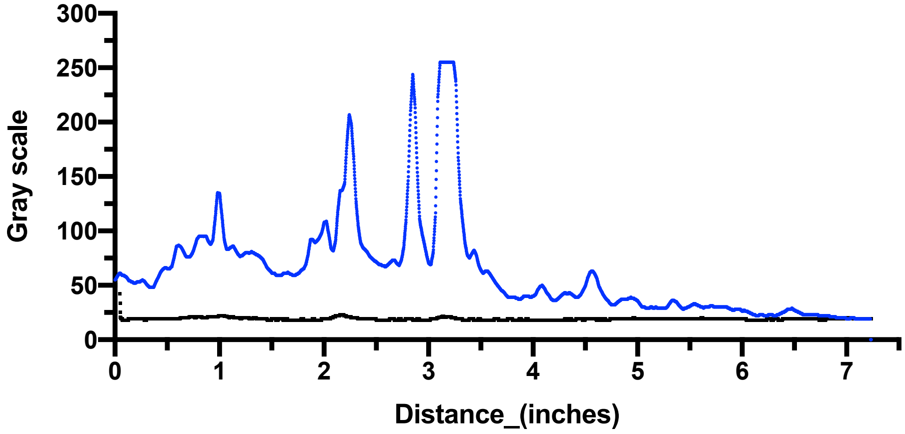

Supplement: Supplementary file 1 [file proteomes-08-00037-s001.zip › Kesari-etal-Supplementary materials/Kesari-etal-Supplementary-File-1.pdf]
